# Limits of Stability during a Therapeutic Exercise Intervention for Instability: Progression, Responders’ and Non-Responders’ Analysis and Predictors

**DOI:** 10.3390/jcm13175036

**Published:** 2024-08-25

**Authors:** Laura Flix-Díez, Melissa Blanco-Pareja, Nicolás Pérez-Fernández

**Affiliations:** Department of Otorhinolaryngology, Clinica Universidad de Navarra, 28027 Madrid, Spain; mblancop@unav.es (M.B.-P.); nperezfer@unav.es (N.P.-F.)

**Keywords:** exercise therapy, instability, limits of stability, rehabilitation, prognosis, physical therapy modalities

## Abstract

**Background/Objectives**: Instability is one of the main symptoms in patients with vestibular and neurological disorders and therapeutic exercise interventions are increasing in popularity as a form of treatment. Additionally, the limits of stability measurement are known to be a good tool for balance evaluation and monitoring of these interventions. The aim of this work is to better understand how a specific protocol provokes changes on this variable and to study the characteristics of those who do and do not respond to it. **Methods**: A retrospective study was developed with the data of 40 patients treated in the Otorhinolaryngology department in Clínica Universidad de Navarra (Madrid, Spain). They had an initial reduction in limits of stability, completed the proposed protocol with home-based and hospital-based exercises and with frequent limits of stability remeasurement, and were assisted to a follow-up retest after 1–2 months. **Results**: A progressive improvement in limits of stability measure was developed through the intervention and was partially retained at follow-up visit. Several differences were found between those patients who improved with the treatment (responders) and those who did not improve (non-responders). More specifically an initial measure of the limits of stability was able to differentiate those groups with a cut-off data of 56 cm^2^. **Conclusions**: The proposed protocol was able to induce motor learning in patients included in this study with good retention after 1–2 months. Furthermore, there is some variability in how patients respond to the treatment. Age and diagnosis should be considered and an interesting cut-off data for clinal decision making was found.

## 1. Introduction

Instability is one of the main symptoms in patients with vestibular and neurological disorders [1,2,3,4]. This condition can be even more significant in the elderly population as general deterioration and comorbidities could have a stronger impact in quality of life and risk of falls [5,6]. This condition has a huge impact on society, making fall prevention one of the main objectives of a healthcare system [6]. Because of this, therapeutic exercise (TE) interventions are increasing in popularity as a form of treatment for these instability issues.

Several types of TE approaches are described according to objectives and targeted population. When addressing vestibular processing, terminology such as vestibular rehabilitation is typically used [7]. On the other hand, for the fall prevention and frailty in the elderly the multicomponent exercise programs (or multicomponent exercise training) are recommended [8]. If the problem or disorder is neurological in nature, neurorehabilitation is utilized [9,10]. However, vestibular rehabilitation is also used in some of these cases [11]. All of these modalities have exercises focused on improving instability. The variability in the literature definitions make interpretation of research and clinical application more difficult [12,13,14].

The intricacy of properly defining and describing rehabilitation [15], underscores the need for specifically delineating the interventions based on their objectives rather than relying on the classifications explained above. Usually, those objectives are set in concordance with different functional tests that show and quantify deficits that can be treated with specific exercises. This approach can help clinicians to develop more individualized TE interventions, which seems to be more similar to the usual physical therapy practice [16] and could lead to better clinical outcomes [17].

There are several different tests aimed at predicting and orienting the appropriate treatment. These provide an adequate assessment of the main functional goals of the balance system, particularly of the ability to maintain body alignment, the facilitation of voluntary movement and reaction in order to recover equilibrium in response to external disturbances [18]. For the las two issues, assessing the limits of stability (LOS) is a good tool, as it determines the ability to dynamically balance weights, i.e., controlling the center of mass with respect to the support base [19].

LOS refers to the area inside which an individual can move their center of mass and maintain equilibrium without changing their base of support [20]. This evaluation can be made by direct measurement, using posturography platforms [21,22,23], using wearable dispositive or by using secondary, but more functional, tests, such as the multi-directional reach test [24,25]. In posturography platforms, data can report different variables, such as the endpoint excursion (point or area) and the maximum excursion (point or area), either as percentages of the theoretical maximum or as the total area measured in square centimeters [21,22,23].

This is a validated variable that, due to its clinical implication, has been studied in different medical conditions such as unilateral vestibulopathy [26], Ménière’s disease [27], Parkinson’s disease [28], multiple sclerosis [29], brain injury [30], and amputations of the lower extremity [31]. Its evaluation shows not just an impairment in some balance capacity, but can also be used as an identifier of fallers in some of those populations [32,33,34,35]. Furthermore, improvement of the LOS can also be associated with a decreased occurrence of falls [34,36].

Furthermore, there is some literature about different types of TE interventions that show an improvement in LOS [17,36,37,38,39]. The main part of these interventions have not been properly defined for replication and data are usually not recorded throughout the process. This makes it difficult to compare different interventions and to properly understand how the improvement occurs. Additionally, it is not known if patients with different characteristics could respond better or worse to those interventions.

Consequently, the aim of this work is to better understand how the improvement of LOS through exercise-based treatment is developed, analyzing the evolution of this variable along the process with a specific intervention created for this purpose. The main hypothesis for this search is that there is a global and progressive increase in LOS measurement because of motor learning processes. Furthermore, there would probably be some participants for whom this intervention will not help to improve their LOS. Consequently, differences between those patients that respond with a good follow up to the treatment and those who do not respond to the protocol will be analyzed. As a final step, this study will search for clinical rules that could help decision making in clinical practice through possible predictors, considering the characteristics of patients that would either respond or not to the specific intervention proposed.

## 2. Materials and Methods

### 2.1. Study Design and Setting

This is a retrospective study made with the data of patients treated with TE from the Otorhinolaryngology department in Clínica Universidad de Navarra (Madrid, Spain) with the objective to improve balance function. All of the patients were seen for instability or dizziness symptoms. Diagnostic criteria for the different disorders were those set as guidelines by the Barany Society International Classification of Vestibular Disorders Consensus Documents easily accessed at https://www.thebaranysociety.org/icvd-consensus-documents (Accessed from November 2021 to May 2024). For the development of this paper the established guides for improving the reporting of TE intervention [40] were used, taking into consideration the STROBE guideline [41] for the global report and the CERT template [42] for the exercise explanation.

Data were collected between November 2021 and May 2024. All of the patients signed an informed consent for the use of their data. This study was conducted in accordance with the ethical standards as laid down in the Declaration of Helsinki and its later amendments.

### 2.2. Participants

The inclusion criteria for the study required participants to show a significant reduction in the LOS at the start of the treatment, taking into consideration the cutoff point assessed by the software platform used for the measurements (Synapsys SPS, INVENTIS S.R.L., Padova, Italy). Exclusion criteria were not following the complete protocol of exercises as indicated during the first visit (due to non-adherence to the treatment, any side effects that necessitated changes to the protocol or rapid improvement) or failing to attend the programmed follow-up visit 1–2 months after the end of the treatment.

### 2.3. Protocol

**Figure 1:**
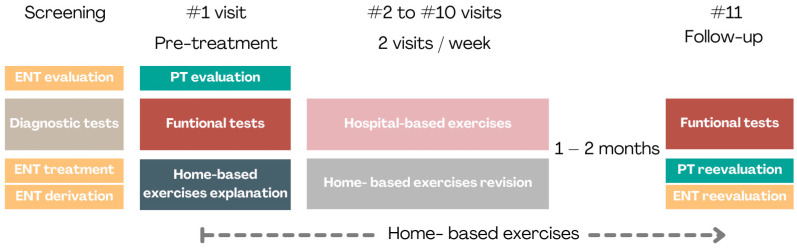
Summary plan of evaluation and treatment in this study. ENT = ear, nose and throat specialist; PT = physical therapist.

The initial evaluation before starting the treatment included specialized vestibular examination by an ear nose and throat (ENT) specialist with long experience in vestibular disorders and further evaluation by the physiotherapist (PT). In the screening with ENT, clinical history and the corresponding diagnostic tests, such as videonistagmography (VNG) or video head impulse tests (vHIT), were properly developed to assess the diagnosis and choose the corresponding treatment for each patient. After this, at the initial PT visit, several functional tests were undertaken to assess the balance capacity of each patient and the patients began a dual working plan (hospital-based and home-based) according to the symptoms and deficits found and the objectives set at that time. This way, participants included in this study could carry out different exercises based on this initial evaluation in addition to the specific exercises designed to improve LOS that are the subject of this work. A final follow-up visit was scheduled in the final session where functional tests were repeated (Figure 1).

**Figure 2:**
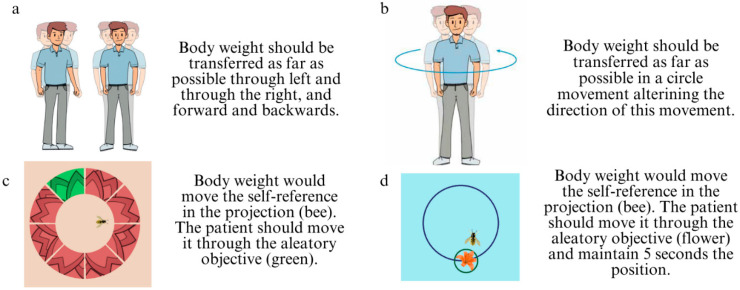
Protocol exercises. (**a**) Home-based exercise with linear translation of body weight transferred as far as possible leftward and rightward as well as forward and backward. (**b**) Home-based exercise with circular translation of body weight in a circular movement in alternate directions. (**c**) Hospital-based exercise set at 90% of the limits of the stability measure. (**d**) Hospital-based exercise set at 70% of the limits of stability measure. ((**c**,**d**) images extracted from Synapsys SPS, INVENTIS S.R.L., Padova, Italy).

The home-based exercises for increasing LOS are shown in Figure 2a,b and were explained by the PT and reviewed twice per week during on-site visits. These were undertaken on firm surface with eyes open, without any necessary material, assuring that in case of marked instability someone familiar was available to help in case of a near fall. Depending on the adherence capacity of the patient the frequency of exercises was 3 or 5 times/day for 1 min/exercise, being always motivated to exercise as many times as possible. No progression was considered in these exercises.

The corresponding hospital-based exercises were performed individually on the posturography platform. The patient was surrounded by a safety bar and a PT, who also taught the exercise, was close to the patient to assist in case of need. For the setting of these exercises a measure of LOS was recorded before beginning each visit. Then, the first (Figure 2c) and the second (Figure 2d) exercises were undertaken at 90% and 70% of that initial measurement, respectively. Both exercises were undertaken for 2 min each one. This method allowed us to set the limits of body displacement increasingly in concordance with the patient’s ability and progression; additionally, it provided a visual reinforcement signal when limits were reached, which could increase the motivation and attentional focus.

### 2.4. Outcome Measures

As a general assessment, the following information from the clinical history was collected: age (years), sex (male/female), and main diagnosis for derivation to vestibular rehabilitation.

The LOS were measured at inclusion, at the beginning of each day of therapy intervention and 1–2 months after ending, with a total of 11 visits (Figure 1). In every measure of LOS the patient was encouraged to displace his/her body weight as far as he/she could: forward, backward, to the right, to the left, in a circle clockwise and in a circle counterclockwise [43]. For those measurements just one trial was undertaken unless the patient failed to properly fulfill the trial by falling, stepping or moving their feet out of the initial position. In this case another trial until was attempted until a correct execution was completed. The total area measured in square centimeters (cm^2^) was recorded.

All intervention and LOS measures were undertaken by the same PT.

### 2.5. Statistical Analysis

Statistical analysis was carried out using STATA 15 (StataCorp. 2017. Stata Statistical Software: Release 15. College Station, TX, USA: StataCorp LLC.)

For descriptive data, quantitative variables were described with mean and standard deviation (SD); frequency and percentage were used to describe qualitative variables. Medical diagnostics were subclassified considering unilateral vestibulopathy, bilateral vestibulopathy or non-objectified vestibular deficit.

Data distribution of the variables was studied with the Shapiro–Wilk test and box plot visualization. Mean differences of quantitative and qualitative variables were compared using student’s *t*-test and Ficher’s tests, respectively, as all variables were normally distributed. When three quantitative groups where compared, a three-way ANOVA was performed. Post hoc comparations were undertaken using the Turkey–Kramer procedure. The criteria of normality and homogeneity of the variances was met.

Patients were classified as responders or non-responders based on the change of the LOS between pre-treatment and final follow up (#1 and #11 visit). This differentiation was undertaken taking into consideration the minimal detectable change of the LOS test [23]. Consequently, patients in the “responders” group had at least an increase in LOS of 27.1 cm^2^ and those in the “non-responders” did not reach that amount of LOS modification.

Finally, to find a cut of data to differentiate responders from non-responders with the initial data of patients, an ROC curve analysis was performed. The higher sensibility and specificity cutoff point was chosen. Finally, for the capacity discrimination of the model the area under de curve (AUC) was analyzed considering the following: AUC equal to 0.50 would indicate no model discrimination, AUC between 0.5 and 0.7 would be poor discrimination, AUC between 0.70 and 0.80 would be acceptable discrimination, AUC between 0.80 and 0.9 would be excellent discrimination and AUC higher than 0.90 would be superior discrimination [44].

To evaluate the magnitude of several studied differences Cohen’s d was calculated and interpreted as the following empirical guideline values for evaluating skills: 0.13 was considered a small effect, 0.28 was considered a medium effect and 0.55 was considered a large effect [45]. Additionally, the confidence interval within 95% (95%CI) for some parameters was calculated for a better understanding of the precision of the data. The threshold for statistical significance was *p* < 0.05.

## 3. Results

A total of 40 patients were included, comprising 26 females (65%) and 14 males (35%), with a mean age of 70.25 years old (SD 12.09 years old). The main medical diagnoses are shown in Table 1.

**Figure 3:**
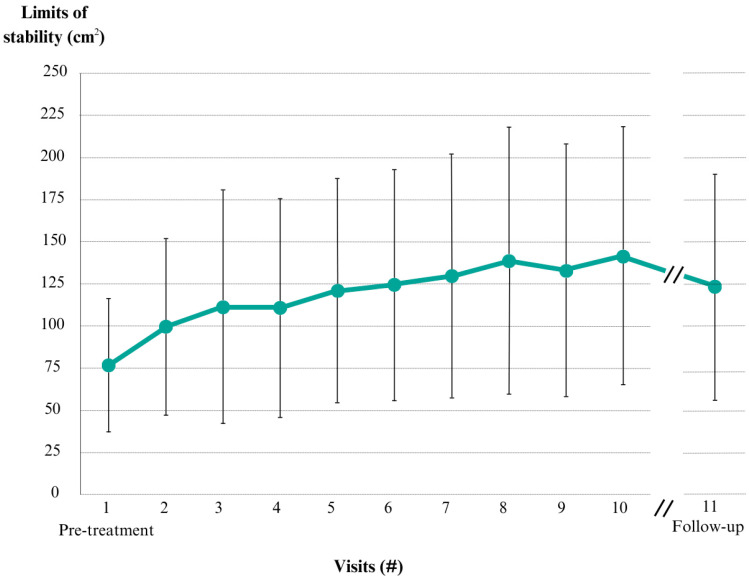
Limits of stability evolution throughout the visit days (cm^2^) for all patients included in the study. Mean of each day and standard deviation with whiskers.

Overall, a significant improvement in LOS was observed across the population throughout the treatment days (#1 with #10 visit, *p* < 0.001) with large effect (Cohen’s d = 1.09; 95%CI 0.61–1.57). This improvement was partially maintained at the re-evaluation visit. Although it was reduced from the last treatment session (#10 with #11 visit, *p* = 0.001) with a small effect (Cohen’s d = 0.23; 95%CI 0.21–0.68), it remained higher than the initial evaluation (#1 with #11 visit, *p* < 0.001) and still had a large global effect (Cohen’s d = 0.9; 95%CI 0.43–1.37). All temporal evolution can be seen in Figure 3 and more specific data in Table 2.

**Figure 4:**
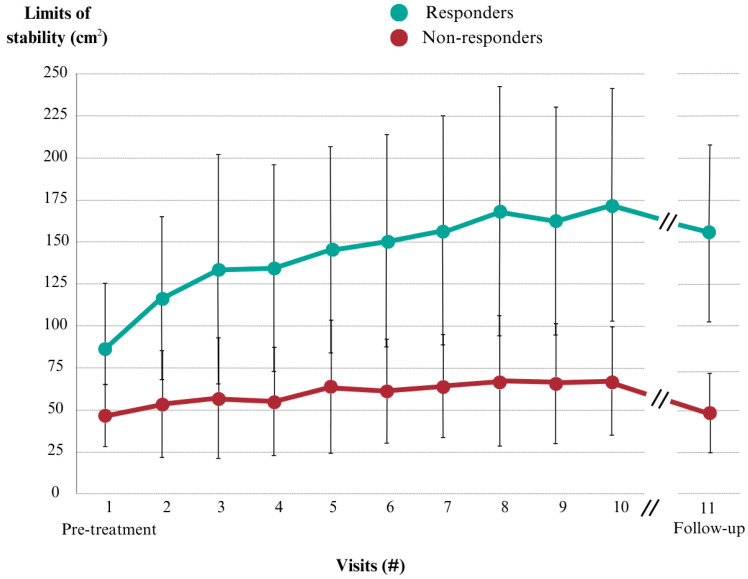
Limits of stability follow-up throughout the visit days in square centimeters (cm^2^) in responder and non-responder groups. Mean of each day and standard deviation with whiskers.

According to the minimal detectable change of LOS measure, 70% of patients (n = 28) were considered as “responders” increasing their LOS area, while 30% (n = 12) as “non-responders,” 11 of them did not improve and in 1 case there was a reduction. This case was a 93-year-old man with presbyvestibulopathy who had an infectious process through the intervention and had to be hospitalized; therefore, the reduction in LOS is considered not to be a consequence of the treatment. This case was deemed an outlier and was excluded from subsequent data analysis. The temporal LOS follow-up with respect to responders vs. non-responders can be seen in Figure 4 and more specific data of the evolution of each group can be found in Table 2.

No differences were found between responders and non-responders for age (*p* = 0.1), gender (*p* = 0.71) and diagnostic group (*p* = 0.26) variables. However, age was closely to be significant as non-responders were slightly older than responders (Table 3). Patients with bilateral vestibulopathy also showed a higher tendency to be non-responders (Table 4).

Significant differences were found in the pre-treatment measurement of LOS (#1 visit) between responders and non-responders (*p* = 0.002) as responders had a higher LOS area (difference of means of 40.38 cm^2^; 95%CI 15.36 cm^2^–65.41 cm^2^). Interestingly, responders also showed a higher increase between the first and second LOS measure (#1 and #2 visit, *p* = 0.03) (difference of means of 22.85 cm^2^; 95%CI 1.56 cm^2^–44.15 cm^2^).

To better understand the influence of those two variables related with the response to treatment, several correlations had been studied. On one hand, pre-treatment LOS measure showed a low correlation (adjusted r^2^ = 0.29; *p* < 0.001) with age, it was not different between sex (*p* = 0.99) within either diagnostic classification (*p* = 0.23). On the other hand, the difference between the first and the second LOS measure (#1 and #2 visit) did not show any correlation with age (*p* = 0.58) or sex (*p* = 0.66). Furthermore, differences between diagnostic classification were found (*p* = 0.007) as, in post hoc analysis, the improvement between the first and second LOS measure (#1 and #2 visit) was found to be higher in unilateral vestibulopathy compared with bilateral vestibulopathy patients (*p* = 0.01; mean difference of 35.32 cm^2^; 95% CI 6.83–63.81) and with non-objectified vestibular deficit (*p* = 0.008; mean difference of 28.81 cm ^2^; 95% CI 7.83–49.80 cm^2^).

**Figure 5:**
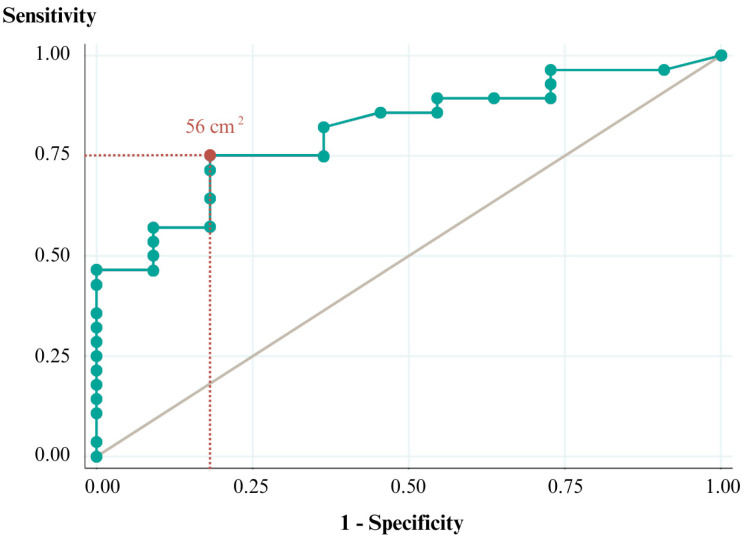
ROC curve to differentiate responders and non-responders to the initial evaluation of limits of stability in centimeters square (cm^2^). The maximum point with the best area under the curve is highlighted in red color.

Finally, a cutoff point in pre-treatment measurement of LOS (#1 visit) was found. An initial LOS measure of 56 cm^2^ was able to differentiate between responders and non-responders with a sensitivity of 75.00% (95%CI 61.41–88.59%) and specificity of 81.82% (95%CI 69.71–93.92%). The area under the ROC curve was 0.81, thus considered an excellent discrimination model (Figure 5).

## 4. Discussion

The TE protocol was able to induce motor learning in patients with instability. This is reflected in the progressive general increase of the LOS measure throughout the process. The selection of these exercises was undertaken considering several aspects of motor learning, aiming to optimize the process as much as possible. The type of exercise is based on the principle of specificity [46,47], and its frequency (especially for exercises performed at home) on the principle of repetition, which facilitates retention [47]. The duration of each exercise was designed to avoid inducing any fatigue that acts as a negative effect on motor learning [48]. Exercises with posturography help to maintain external attentional focus and frequent rewards by providing visual feedback, which is positive for motor learning [49,50]. The distance of displacement when performing the posturography exercises in different sessions was conveniently modified so as to help to develop an individualized and progressive protocol over a period of days, supporting the principle of intensity and making the exercises consistently challenging [47].

The frequency of on-site sessions in the hospital is sufficient to produce a positive summating effect, as seen by the progressive increase in LOS throughout the treatment. Additionally, the total number of visits is enough to start producing some ceiling effect as the differences between the initial sessions are higher than those between the latest. As seen in Figure 3, there is a ceiling effect at visit 8 that questions the idea of the correct number of sessions needed in a clinical environment; for this, another study must be undertaken considering the specific disorder, the patient’s natural history and their age, aspects that affect the individual variability of the velocity of motor learning [47,51,52,53].

We demonstrate that final retention was obtained, as indicated by the difference between LOS measures in the first follow-up, undertaken 1–2 months after ending the treatment. There is a partial loss of improvement for a measure when it is compared with the end-of treatment result that we follows months later. As such home exercises do not seem sufficient to maintain the improvement obtained during the presential sessions. Unfortunately, no report of the adherence to the treatment was recorded. This adherence, or even the amount of daily activity that the patients undertook during this period, could affect this process given the principle of “use it or lose it” [47] and should be recorded in further studies.

There is some variability in how patients respond to TE treatment. There is a tendency of the non-responder group to be older than responders, an expected result due to the deleterious effect of age on learning [47,53]. Additionally, responders showed a faster improvement, as shown by the difference in LOS between visits 1 and 2; this difference sems to be related mainly to unilateral vestibulopathy patients, and as some were included in a sub-acute state, the natural follow-up could have influence in this result. However, we have not seen a major difference in results according to diagnosis. Finally, an unexpected finding was that the initial LOS area helps to differentiate responders from non-responders and that a cutoff data of <56 cm^2^ worked as a predictor for the follow-up with relatively good sensibility and specificity.

All of these results show an interesting starting point to analyze how patients respond to treatments that are focused on improving LOS and improve protocols taken in consideration of the individual characteristics of the patients. This way it would be interesting to compare the results with other types of interventions and clinical contexts as this study also has several limitations. We did not compare patients with other types of interventions or with a control group and data can also be influenced by natural history, main regression, placebo effects, specific characteristics of our clinical context or retrospective analysis. Considering this, it is possible that the cut-off point could change or at least could lead to improvements in its sensitivity and specificity. Finally, to better understand the clinical implication of these protocols, the changes of LOS measure should be studied in correlation with other clinical outcomes, such as patient-reported outcomes or other balance and clinical tests, as well as risk of fall evaluations [32,33,34,35,36,54].

## 5. Conclusions

The TE protocol was able to induce motor learning in patients with instability with good retention after 1 or 2 months, while maintaining home-based exercises. Furthermore, there is some variability in how patients respond to the treatment. Age and diagnosis should be considered when analyzing these temporal differences. Interestingly, initial LOS area helps to differentiate responders form non-responders with a cutoff data of <56 cm^2^, which could help us in a clinical environment by acting as a predictor to improve clinical decision making.

## Figures and Tables

**Table 1:** Descriptive data of the main diagnosis of the patients included in the study. BPPV = benign paroxysmal positional vertigo; SSCD = superior semicircular canal dehiscence; PPPD = perceptual postural persistent dizziness.

Diagnosis of the Patients Included in the Study.		
Unilateral vestibulopathy	32.50% (n = 13)	Acute (n = 4) Acute in evolution (n = 2) Chronic (n = 7): after AUV (3), after gentamicin treatment for Ménière’s disease (3) and, with combined BPPV (1).
Bilateral vestibulopathy	27.50% (n = 11)	Definite: idiopathic (n = 7) Probable (n = 1) Presbyvestibulopathy “definite” (n = 1) Added to cerebellar ataxia (n = 1) or Parkinson’s disease (n = 1)
Non-objectified vestibular deficit	40% (n = 16)	Non-specific instability (n = 6) Residual dizziness to BPPV (n = 2) BPPV “probable” (n = 2) SSCD (n = 2) Chronic stroke (n = 1) PPPD “probable” with down beat nystagmus syndrome (n = 1) Vestibular migraine “definite” (n = 1) Drugs side effects (n = 1)

**Table 2:** Limits of stability (LOS) evolution through the days in centimeters square (cm^2^) in the responder and non-responder groups. Mean of each day with standard deviation (SD).

	Total of Participants (n = 40)	Responders (n = 28)	Non-Responders (n = 11)
Pre-treatment LOS #1	76.8 cm^2^ (SD 39.45)	86.75 cm^2^ (SD 38.98)	46.36 cm^2^ (SD 18.80)
LOS #2	99.47 cm^2^ (SD 52.27)	116.47 cm^2^ (SD 48.54)	53.22 cm^2^ (SD 32.04)
LOS #3	111.55 cm^2^ (SD 69.02)	133.64 cm^2^ (SD 68.33)	56.92 cm^2^ (SD 35.93)
LOS #4	110.94 cm^2^ (SD 64.99)	134.38 cm^2^ (SD 61.75)	55.06 cm^2^ (SD 32.24)
LOS #5	121.07 cm^2^ (SD 66.27)	145.28 cm^2^ (SD 61.13)	63.92 cm^2^ (SD 39.53)
LOS #6	124.49 cm^2^ (SD 68.39)	150.74 cm^2^ (SD 63.24)	61.22 cm^2^ (SD 30.93)
LOS #7	129.80 cm^2^ (SD 72.29)	156.92 cm^2^ (SD 68.04)	64.33 cm^2^ (SD 30.84)
LOS #8	138.77 cm^2^ (SD 79.42)	168.38 cm^2^ (SD 74.1)	67.5 cm^2^ (SD 39.06)
LOS #9	133.1 cm^2^ (SD 74.95)	162.66 cm^2^ (SD 67.86)	65.76 cm^2^ (SD 35.79)
LOS #10	141.76 cm^2^ (SD 76.48)	172.17 cm^2^ (SD 69.28)	67.11 cm^2^ (SD 32.33)
Follow-up LOS #11	123.75 cm^2^ (SD 67.55)	155.85 cm^2^ (SD 53.14)	48.00 cm^2^ (SD 23.27)

**Table 3:** Descriptive data of the age (years old as y.o.) of patients classified as responders and non-responders to the treatment. Mean and standard deviation (SD) with its corresponding confidence interval within 95% (95%CI).

	Mean	SD	95% CI
Responders (n = 28)	67.78 y.o.	11.61	63.28–72.29 y.o.
Non-responders (n = 11)	74.45 y.o.	10.83	67.17–81.73 y.o.
Difference between both groups	6.66 y.o.		−1.55–14.89 y.o.

**Table 4:** Descriptive data of the patients according to diagnostic classification and response to the treatment. Absolute number and percentage in brackets.

	Responders (n = 28)	Non-Responders (n = 11)
Unilateral vestibulopathy	10 (76.92%)	3 (23.08%)
Bilateral vestibulopathy	5 (50%)	5 (50%)
Non-objectified vestibular deficit	13 (81.25%)	3 (18.75%)

## Data Availability

The data presented in this study are available on request from the corresponding author. (The data are not publicly available due to patients’ privacy).
